# Di-μ-methacrylato-κ^4^ *O*:*O*′-bis­[aqua­bis(1,10-phenanthroline-κ^2^ *N*,*N*′)copper(II)] dinitrate dihydrate

**DOI:** 10.1107/S1600536808016218

**Published:** 2008-06-07

**Authors:** M. T. H. Tarafder, M. Y. Reza, K. A. Crouse, Suchada Chantrapromma, Hoong-Kun Fun

**Affiliations:** a Department of Chemistry, Rajshahi University, Rajshahi 6205, Bangladesh; b Department of Chemistry, Universiti Putra Malaysia, 43400 Serdang, Selangor, Malaysia; c Department of Chemistry, Faculty of Science, Prince of Songkla University, Hat-Yai, Songkhla 90112, Thailand; d X-ray Crystallography Unit, School of Physics, Universiti Sains Malaysia, 11800 USM, Penang, Malaysia

## Abstract

The title complex, [Cu_2_(C_4_H_5_O_2_)_2_(C_12_H_8_N_2_)_2_(H_2_O)_2_](NO_3_)_2_·2H_2_O, contains a dimeric [Cu_2_(C_4_H_5_O_2_)_2_(C_12_H_8_N_2_)_2_(H_2_O)_2_]^2+^ dication with two five-coordinated Cu^II^ ions linked by two methacrylate ions in a *syn*–*syn* bridging arrangement. The dication possesses pseudo-twofold rotational symmetry. The penta­coordination of each Cu^II^ ion has a distorted square-pyramidal geometry, with two N donors from a phenanthroline ligand and two carboxyl­ate O atoms occupying basal sites and the apical position being occupied by a water mol­ecule. In the crystal packing, mol­ecules are linked to form a three-dimensional framework by O—H⋯O and C—H⋯O hydrogen bonds and π–π inter­actions [centroid–centroid distances of 3.6039 (15),  3.5301 (15),  3.6015 (15), 3.6496 (15) and  3.6858 (15) Å].

## Related literature

For bond-length data, see: Allen *et al.* (1987 ▶). For structures of related copper(II) complexes, see: Chen *et al.* (2008 ▶); Perlepes *et al.* (1995 ▶). For related literature, see: Besecke *et al.* (1989 ▶); Blackburn *et al.* (1995 ▶); Chen *et al.* (2007 ▶); Dang (1994 ▶); Houser *et al.* (1996 ▶); Matsushima *et al.* (1995 ▶); Reza *et al.* (1998 ▶, 1999 ▶, 2003 ▶); Tokii *et al.* (1989 ▶, 1990 ▶, 1992 ▶, 1995 ▶); Schubert (1996 ▶); Schubert *et al.* (1992 ▶, 1995 ▶).
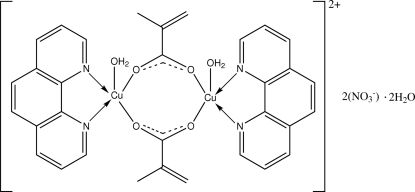


## Experimental

### 

#### Crystal data

[Cu_2_(C_4_H_5_O_2_)_2_(C_12_H_8_N_2_)_2_(H_2_O)_2_](NO_3_)_2_·2H_2_O
*M* *_r_* = 853.75
Monoclinic, 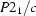

*a* = 13.6146 (2) Å
*b* = 15.7322 (2) Å
*c* = 16.4463 (2) Å
β = 102.1306 (8)°
*V* = 3443.94 (8) Å^3^
*Z* = 4
Mo *K*α radiation
μ = 1.31 mm^−1^
*T* = 100.0 (1) K
0.27 × 0.24 × 0.16 mm

#### Data collection

Bruker SMART APEXII CCD area-detector diffractometer
Absorption correction: multi-scan (*SADABS*; Bruker, 2005 ▶) *T*_min_ = 0.716, *T*_max_ = 0.822
43546 measured reflections
10036 independent reflections
6885 reflections with *I* > 2σ(*I*)
*R*_int_ = 0.069

#### Refinement

*R*[*F* ^2^ > 2σ(*F* ^2^)] = 0.047
*wR*(*F* ^2^) = 0.113
*S* = 1.04
10036 reflections
489 parameters
H-atom parameters constrained
Δρ_max_ = 0.68 e Å^−3^
Δρ_min_ = −0.78 e Å^−3^

### 

Data collection: *APEX2* (Bruker, 2005 ▶); cell refinement: *APEX2*; data reduction: *SAINT* (Bruker, 2005 ▶); program(s) used to solve structure: *SHELXTL* (Sheldrick, 2008 ▶); program(s) used to refine structure: *SHELXTL*; molecular graphics: *SHELXTL*; software used to prepare material for publication: *SHELXTL* and *PLATON* (Spek, 2003 ▶).

## Supplementary Material

Crystal structure: contains datablocks global, I. DOI: 10.1107/S1600536808016218/ci2604sup1.cif

Structure factors: contains datablocks I. DOI: 10.1107/S1600536808016218/ci2604Isup2.hkl

Additional supplementary materials:  crystallographic information; 3D view; checkCIF report

## Figures and Tables

**Table 1:** Hydrogen-bond geometry (Å, °)

*D*—H⋯*A*	*D*—H	H⋯*A*	*D*⋯*A*	*D*—H⋯*A*
O1*W*—H1*W*1⋯O6^i^	0.85	2.42	3.115 (3)	140
O1*W*—H1*W*1⋯O8^i^	0.85	2.32	2.882 (3)	124
O2*W*—H1*W*2⋯O5^ii^	0.85	2.03	2.761 (3)	144
O3*W*—H1*W*3⋯O5	0.86	1.97	2.811 (3)	163
O4*W*—H1*W*4⋯O10^iii^	0.93	2.02	2.807 (3)	142
O1*W*—H2*W*1⋯O3*W*^i^	0.85	2.19	2.791 (3)	127
O3*W*—H2*W*3⋯O9^iii^	0.91	2.00	2.862 (3)	157
O4*W*—H2*W*4⋯O7^iii^	0.84	2.29	2.860 (3)	125
C1—H1*A*⋯O4	0.93	2.56	3.035 (3)	112
C1—H1*A*⋯O10^iv^	0.93	2.53	3.247 (3)	134
C3—H3*A*⋯O9^v^	0.93	2.37	3.186 (4)	146
C14—H14*A*⋯O4*W*^vi^	0.93	2.52	3.364 (4)	151
C15—H15*A*⋯O2*W*^ii^	0.93	2.49	3.357 (3)	155
C21—H21*A*⋯O3^v^	0.93	2.39	3.318 (4)	179
C28—H28*B*⋯O3	0.93	2.42	2.747 (4)	100
C32—H32*B*⋯O1	0.93	2.42	2.737 (4)	100
C32—H32*B*⋯O8^i^	0.93	2.43	3.345 (3)	168

Symmetry codes: (i) 
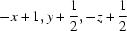
; (ii) 
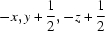
; (iii) 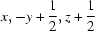; (iv) 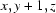; (v) 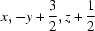; (vi) 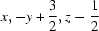.

**Fig. 1.**
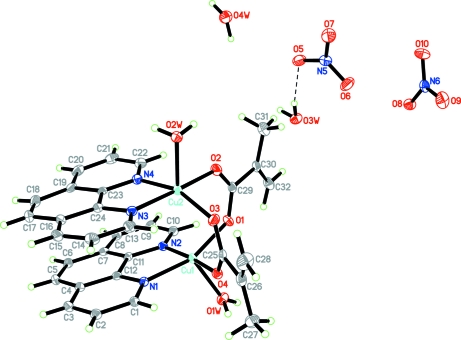
The asymmetric unit of the title compound, showing 50% probability displacement ellipsoids and the atomic numbering. The O—H···O hydrogen bond is shown as a dashed line.

**Fig. 2.**
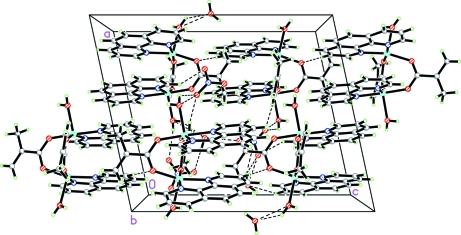
The crystal packing of the title compound, viewed approximately along the b axis. Hydrogen bonds are shown as dashed lines.

## supplementary crystallographic information

### Comment

There is considerable interest in bioinorganic chemistry of metal carboxylates as these are formally analogous to organic esters (Dang, 1994; Reza *et al.*, 2003). In this type of complex, the reactivity of the carboxylates towards the nucleophiles is enhanced (Houser *et al.*, 1996; Blackburn *et al.*, 1995; Reza *et al.*, 1998; 1999; Tokii *et al.*, 1989). Since transition metal complexes of methacrylic acid are also polymeric (Schubert, 1996; Schubert *et al.*, 1992, 1995), chemists are attracted to study the application of these types of materials, particularly as catalysts. The Cu^II^ ions coordinate with a variety of carboxylates (Besecke *et al.*, 1989; Matsushima *et al.*, 1995). Such related coordinations have appeared in a series of binuclear Cu^II^ complexes with 1,3-bis(hydroxyphenyl)-2-imidazolidinethione, [Cu(RCOO)(H*L*^1^)]_2_ (*R* = CH_3_, C_6_H_5_),(H*L*^1^= 1-hydroxymethyl-3-methyl-2-imidazolidinethione), [Cu(RCOO)(*L*^2^)]_2_ (*R* = CH_3_, 2-CH_3_C_6_H_4_, and 4-CH_3_C_6_H_4_) (imidazolidinethione being the second substituent of the aryl ring) (Tokii *et al.*, 1995), bis(µ-carboxylato-O,*O*')-diaquobis (1,10-pherathroline) dicopper(II) dinitrate tetrahydrates [Cu(RCOO)(phen)(H_2_O)]_2_(NO_3_)_2_. 4H_2_O [*R* = H, CH_3_ and (CH_3_)_3_C] (Tokii *et al.*, 1990; 1992). Matsushima *et al.* (1995) have reported some triply bridged dinuclear carboxylato copper (II) complexes, [Cu_2_(Ph_2_CHCOO)_3_(*L*)_2_]BF4 [*L* = 2.2'-bipyridine and 1,10-phenanthroline]. From these related coordinations (Perlepes *et al.*, 1995), we found there is no report on conjugated double-bond systems containing a monobasic acid (*e.g.* methacrylic acid) with *syn*–*syn* bridging modes of binuclear Cu(II) and this has prompted us to attempt to prepare a binuclear Cu(II) complex with phenanthroline (phen) and methacrylic acid. Methacrylic acid and phenanthroline were used to gain some insight into the flexiblity of these complexes and also the effect of these auxiliary ligands on stacking. We report herein the first example of a binuclear Cu^II^ complex of this type, [Cu(C_3_H_5_COO)(phen)(H_2_O)]_2_(NO_3_)_2_.2H_2_O, along with its crystal structure.

The asymmetric unit of the title compound consists of a dinuclear [Cu(C_3_H_5_COO)(phen)(H_2_O)]_2_^2+^ cation, two NO_3_^-^ anions and two H_2_O molecules (Fig. 1). The coordination environment of each Cu^II^ ion is CuN_2_O_3_ in which the basal positions are formed by two N atoms from a bidentate phenanthroline ligand [Cu1—N1 = 2.014 (2) Å, Cu1—N2 = 2.018 (2) Å and Cu2—N3 = 2.008 (2) Å, Cu2—N4 = 2.019 (2) Å] and two O atoms of two bridging methacrylato ligands [Cu1—O1 = 1.9641 (19) Å, Cu1—O4 = 1.9446 (18) Å and Cu2—O2 = 1.9440 (19) Å, Cu2—O3 = 1.956 (2) Å]. The two carboxylate groups are in the bidentate *syn*–*syn* bridging mode. The apical position of each Cu^II^ is occupied by an O atom of a water molecule [Cu1—O1W = 2.1525 (19) Å and Cu2—O2W = 2.1538 (18) Å]. These axial bonds are longer than the bond lengths in the basal positons. Coordination of the N_2_ chelate phenanthroline ligand to the Cu^II^ ion results in the formation of two planar five-membered rings Cu1/N1/N2/C11/C12 (with a maximum deviation of -0.019 (1) Å for atom Cu1) and Cu2/N3/N4/C23/C24 (with a maximum deviation of 0.014 (3) Å for atom C23). The dihedral angle between these two five-membered rings is 5.48 (10)°. The Cu1···Cu2 distance is 3.106 (1) Å. The orientation of the two bridging methacrylato ligands can be indicated by the dihedral angle between the mean planes through Cu1/O3/O4/C25 and Cu2/O1/O2/C29 of 71.74 (13)°. The electron delocalizations in the two carboxylate fragments are complete as can be indicated by the almost equal C—O bond lengths [C25—O3 = 1.263 (3) Å, C25—O4 = 1.261 (3) Å and C29—O1 = 1.259 (3) Å, C29—O2 = 1.266 (3) Å]. All bond lengths are in agreement with other related structures (Chen *et al.*, 2008; Perlepes *et al.*, 1995) and are in normal ranges (Allen *et al.*, 1987).

The two phen ligands of the dinuclear complex are stacked with their centroids separated by 3.625 (1) Å indicating significant π–π interactions. The various centroid–centroid separations involving the two phen ligands are: Cg1···Cg3 = 3.6039 (15)Å, Cg1···Cg6 = 3.5301 (15)Å, Cg2···Cg4 = 3.6015 (15)Å, Cg4···Cg5 = 3.6496 (15)Å and Cg5···Cg6 = 3.6858 (15)Å (Cg1, Cg2, Cg3, Cg4, Cg5 and Cg6 are the centroids of the N1/C1–C4/C12, N2/C7–C11, N3/C13–C16/C24, N4/C19–C23, C4–C7/C11/C12 and C16–C19/C23/C24 rings, respectively).

O—H···O hydrogen bonds between water molecules and the nitrate ions play an important role in stabilizing the crystal structure (Table 1). These hydrogen bonds link the complex molecules, water molecules and nitrate groups into a two-dimensional network parallel to the (010) plane. The two-dimensional network is further strengthened by π–π interactions between two symmetry related C4–C7/C11/C12 rings at (x, y, z) and (1-x, 2-y, 1-z), with their centroids separated by 3.5381 (15) Å. The adjacent two-dimensional network are cross-linked along the *b* axis via weak C—H···O interactions.

### Experimental

The title compound was synthesized by adding a mixture of methacrylic acid (10 mmol) and 1,10-phenanthroline (10 mmol) in water (60 ml) with triethylamine (10 mmol) to aqueous Cu(NO_3_)_2_ (2.42 g, 10 mmol) in water (20 ml) while stirring. The stirring was continued for another half an hour. Precipitates initially formed were filtered and the filtrate was concentrated to one-third of its original volume (25 ml). Deep blue single crystals of the title compound which appeared after a week were collected, washed with water and dried in air at room temperature (m.p. 494 K).

### Refinement

H atoms attached to O atoms (water) were located in difference Fourier maps and constrained to ride on their parent atoms, with U_iso_(H) = 1.2U_eq_(O). C-bound H atoms were placed in calculated positions (C—H = 0.93–0.96 Å) and allowed to ride on their parent atoms, with *U*_iso_ = 1.2-1.5*U*_eq_(C). A rotating group model was used for the methyl groups.

### Crystal data

**Table:**

[Cu_2_(C_4_H_5_O_2_)_2_(C_12_H_8_N_2_)_2_(H_2_O)_2_](NO_3_)_2_·2H_2_O	*F*_000_ = 1752
*M**_r_* = 853.75	*D*_x_ = 1.647 Mg m^−^^3^
Monoclinic, *P*2_1_/*c*	Melting point: 494 K
Hall symbol: -P 2ybc	Mo *K*α radiation λ = 0.71073 Å
*a* = 13.6146 (2) Å	Cell parameters from 10036 reflections
*b* = 15.7322 (2) Å	θ = 1.5–30.0º
*c* = 16.4463 (2) Å	µ = 1.32 mm^−^^1^
β = 102.1306 (8)º	*T* = 100.0 (1) K
*V* = 3443.94 (8) Å^3^	Block, blue
*Z* = 4	0.27 × 0.24 × 0.16 mm

### Data collection

**Table:**

Bruker SMART APEXII CCD area-detector diffractometer	10036 independent reflections
Radiation source: fine-focus sealed tube	6885 reflections with *I* > 2σ(*I*)
Monochromator: graphite	*R*_int_ = 0.069
Detector resolution: 8.33 pixels mm^-1^	θ_max_ = 30.0º
*T* = 100.0(1) K	θ_min_ = 1.5º
ω scans	*h* = −19→19
Absorption correction: multi-scan(SADABS; Bruker, 2005)	*k* = −21→22
*T*_min_ = 0.716, *T*_max_ = 0.822	*l* = −23→22
43546 measured reflections	

### Refinement

**Table:**

Refinement on *F*^2^	Secondary atom site location: difference Fourier map
Least-squares matrix: full	Hydrogen site location: inferred from neighbouring sites
*R*[*F*^2^ > 2σ(*F*^2^)] = 0.047	H-atom parameters constrained
*wR*(*F*^2^) = 0.113	*w* = 1/[σ^2^(*F*_o_^2^) + (0.0457*P*)^2^ + 1.7397*P*] where *P* = (*F*_o_^2^ + 2*F*_c_^2^)/3
*S* = 1.04	(Δ/σ)_max_ = 0.001
10036 reflections	Δρ_max_ = 0.68 e Å^−^^3^
489 parameters	Δρ_min_ = −0.78 e Å^−^^3^
Primary atom site location: structure-invariant direct methods	Extinction correction: none

### Special details

**Table:**

Experimental. The low-temperature data was collected with the Oxford Cyrosystem Cobra low-temperature attachment.
Geometry. All e.s.d.'s (except the e.s.d. in the dihedral angle between two l.s. planes) are estimated using the full covariance matrix. The cell e.s.d.'s are taken into account individually in the estimation of e.s.d.'s in distances, angles and torsion angles; correlations between e.s.d.'s in cell parameters are only used when they are defined by crystal symmetry. An approximate (isotropic) treatment of cell e.s.d.'s is used for estimating e.s.d.'s involving l.s. planes.
Refinement. Refinement of *F*^2^ against ALL reflections. The weighted *R*-factor *wR* and goodness of fit *S* are based on *F*^2^, conventional *R*-factors *R* are based on *F*, with *F* set to zero for negative *F*^2^. The threshold expression of *F*^2^ > σ(*F*^2^) is used only for calculating *R*-factors(gt) *etc*. and is not relevant to the choice of reflections for refinement. *R*-factors based on *F*^2^ are statistically about twice as large as those based on *F*, and *R*- factors based on ALL data will be even larger.

### Fractional atomic coordinates and isotropic or equivalent isotropic displacement parameters (Å^2^)

**Table:**

	*x*	*y*	*z*	*U*_iso_*/*U*_eq_	
Cu1	0.38427 (2)	0.86242 (2)	0.280115 (19)	0.01328 (8)	
Cu2	0.15436 (2)	0.83947 (2)	0.21652 (2)	0.01440 (9)	
O1	0.36221 (13)	0.74006 (12)	0.26072 (11)	0.0175 (4)	
O2	0.19581 (14)	0.72098 (12)	0.22433 (12)	0.0210 (4)	
O3	0.19962 (13)	0.86057 (13)	0.11307 (12)	0.0195 (4)	
O4	0.36133 (13)	0.89340 (12)	0.16317 (11)	0.0163 (4)	
O1W	0.54224 (14)	0.84782 (13)	0.28332 (12)	0.0206 (4)	
H1W1	0.5927	0.8148	0.2920	0.031*	
H2W1	0.5466	0.8796	0.2424	0.031*	
O2W	0.00149 (13)	0.80624 (12)	0.16106 (12)	0.0189 (4)	
H1W2	−0.0256	0.8171	0.2020	0.023*	
H2W2	0.0072	0.7524	0.1618	0.023*	
N1	0.35977 (15)	0.98300 (14)	0.31179 (13)	0.0136 (4)	
N2	0.40285 (15)	0.84520 (14)	0.40400 (13)	0.0133 (4)	
N3	0.11389 (15)	0.96185 (14)	0.22068 (13)	0.0148 (5)	
N4	0.15054 (15)	0.84559 (14)	0.33844 (14)	0.0147 (5)	
C1	0.33896 (18)	1.05166 (17)	0.26328 (16)	0.0148 (5)	
H1A	0.3400	1.0470	0.2071	0.018*	
C2	0.31551 (19)	1.13053 (18)	0.29446 (17)	0.0174 (6)	
H2A	0.3001	1.1768	0.2589	0.021*	
C3	0.31543 (19)	1.13909 (18)	0.37706 (17)	0.0177 (6)	
H3A	0.2990	1.1909	0.3979	0.021*	
C4	0.34054 (18)	1.06858 (17)	0.43076 (16)	0.0156 (5)	
C5	0.34706 (19)	1.07128 (18)	0.51869 (17)	0.0176 (6)	
H5A	0.3327	1.1217	0.5433	0.021*	
C6	0.37387 (19)	1.00148 (18)	0.56700 (17)	0.0170 (6)	
H6A	0.3791	1.0052	0.6242	0.020*	
C7	0.39418 (18)	0.92214 (17)	0.53086 (16)	0.0149 (5)	
C8	0.42171 (19)	0.84690 (18)	0.57659 (17)	0.0175 (6)	
H8A	0.4275	0.8463	0.6340	0.021*	
C9	0.43985 (19)	0.77448 (18)	0.53534 (16)	0.0171 (6)	
H9A	0.4590	0.7247	0.5649	0.021*	
C10	0.42949 (19)	0.77571 (18)	0.44871 (17)	0.0173 (6)	
H10A	0.4417	0.7261	0.4217	0.021*	
C11	0.38645 (18)	0.91764 (17)	0.44477 (16)	0.0133 (5)	
C12	0.36170 (18)	0.99215 (17)	0.39418 (16)	0.0137 (5)	
C13	0.0902 (2)	1.01713 (18)	0.15840 (17)	0.0185 (6)	
H13A	0.0966	1.0007	0.1054	0.022*	
C14	0.0561 (2)	1.09905 (18)	0.16996 (18)	0.0217 (6)	
H14A	0.0388	1.1358	0.1249	0.026*	
C15	0.0481 (2)	1.12505 (18)	0.24745 (19)	0.0212 (6)	
H15A	0.0258	1.1796	0.2556	0.025*	
C16	0.07407 (19)	1.06854 (17)	0.31525 (17)	0.0169 (6)	
C17	0.0720 (2)	1.08926 (19)	0.39989 (18)	0.0223 (6)	
H17A	0.0540	1.1438	0.4128	0.027*	
C18	0.0960 (2)	1.03040 (19)	0.46151 (18)	0.0220 (6)	
H18A	0.0961	1.0459	0.5161	0.026*	
C19	0.12109 (19)	0.94527 (19)	0.44389 (16)	0.0173 (6)	
C20	0.1392 (2)	0.8790 (2)	0.50233 (18)	0.0215 (6)	
H20A	0.1367	0.8894	0.5575	0.026*	
C21	0.1606 (2)	0.7989 (2)	0.47812 (18)	0.0218 (6)	
H21A	0.1711	0.7547	0.5165	0.026*	
C22	0.16637 (19)	0.78423 (18)	0.39568 (17)	0.0191 (6)	
H22A	0.1818	0.7298	0.3801	0.023*	
C23	0.12645 (18)	0.92432 (17)	0.36181 (17)	0.0145 (5)	
C24	0.10448 (18)	0.98683 (17)	0.29791 (16)	0.0153 (5)	
C25	0.28327 (19)	0.89118 (17)	0.10616 (16)	0.0149 (5)	
C26	0.2907 (2)	0.92989 (18)	0.02407 (17)	0.0205 (6)	
C27	0.3878 (2)	0.9638 (2)	0.01568 (19)	0.0278 (7)	
H27A	0.3827	0.9857	−0.0395	0.042*	
H27B	0.4372	0.9193	0.0257	0.042*	
H27C	0.4075	1.0086	0.0554	0.042*	
C28	0.2060 (2)	0.9363 (2)	−0.03626 (18)	0.0335 (8)	
H28A	0.2080	0.9634	−0.0861	0.040*	
H28B	0.1459	0.9136	−0.0277	0.040*	
C29	0.28507 (19)	0.69382 (17)	0.24574 (16)	0.0154 (5)	
C30	0.2994 (2)	0.59990 (17)	0.25582 (16)	0.0164 (5)	
C31	0.2129 (2)	0.54491 (19)	0.23257 (18)	0.0246 (7)	
H31A	0.2294	0.4892	0.2550	0.037*	
H31B	0.1938	0.5415	0.1730	0.037*	
H31C	0.1581	0.5675	0.2542	0.037*	
C32	0.3946 (2)	0.56991 (19)	0.28914 (17)	0.0225 (6)	
H32A	0.4054	0.5119	0.2975	0.027*	
H32B	0.4480	0.6077	0.3032	0.027*	
N5	0.15097 (17)	0.32307 (16)	0.18469 (15)	0.0220 (5)	
O5	0.15892 (15)	0.33112 (15)	0.26311 (13)	0.0296 (5)	
O6	0.22735 (16)	0.32020 (17)	0.15650 (14)	0.0374 (6)	
O7	0.06546 (15)	0.31781 (15)	0.13950 (14)	0.0326 (5)	
N6	0.33329 (18)	0.17479 (15)	0.07401 (15)	0.0201 (5)	
O8	0.40081 (14)	0.18673 (13)	0.13715 (12)	0.0237 (5)	
O9	0.34045 (18)	0.20737 (15)	0.00610 (13)	0.0348 (6)	
O10	0.25858 (15)	0.13046 (14)	0.07883 (13)	0.0280 (5)	
O3W	0.35788 (15)	0.35991 (13)	0.34804 (13)	0.0242 (5)	
H1W3	0.2960	0.3622	0.3220	0.036*	
H2W3	0.3448	0.3527	0.3996	0.036*	
O4W	0.07983 (16)	0.28832 (15)	0.50131 (13)	0.0319 (5)	
H1W4	0.1462	0.3043	0.5052	0.048*	
H2W4	0.0414	0.2807	0.5347	0.048*	

### Atomic displacement parameters (Å^2^)

**Table:**

	*U*^11^	*U*^22^	*U*^33^	*U*^12^	*U*^13^	*U*^23^
Cu1	0.01626 (15)	0.01130 (17)	0.01213 (16)	0.00004 (12)	0.00262 (12)	−0.00018 (12)
Cu2	0.01577 (15)	0.01211 (17)	0.01579 (17)	0.00068 (12)	0.00441 (12)	−0.00054 (13)
O1	0.0210 (9)	0.0106 (10)	0.0202 (10)	−0.0009 (7)	0.0028 (8)	−0.0017 (8)
O2	0.0203 (9)	0.0144 (10)	0.0288 (11)	0.0017 (8)	0.0057 (8)	−0.0023 (8)
O3	0.0176 (9)	0.0250 (11)	0.0166 (10)	−0.0012 (8)	0.0055 (7)	−0.0027 (8)
O4	0.0201 (9)	0.0144 (10)	0.0144 (9)	−0.0011 (7)	0.0038 (7)	−0.0001 (7)
O1W	0.0172 (9)	0.0251 (12)	0.0193 (10)	0.0037 (8)	0.0035 (8)	0.0022 (8)
O2W	0.0204 (9)	0.0161 (10)	0.0215 (10)	−0.0014 (8)	0.0070 (8)	−0.0008 (8)
N1	0.0142 (10)	0.0125 (11)	0.0148 (11)	0.0001 (8)	0.0047 (8)	0.0001 (9)
N2	0.0132 (10)	0.0118 (11)	0.0151 (11)	0.0000 (8)	0.0032 (8)	0.0010 (9)
N3	0.0140 (10)	0.0137 (12)	0.0173 (11)	0.0002 (9)	0.0048 (8)	0.0025 (9)
N4	0.0141 (10)	0.0141 (12)	0.0160 (11)	0.0011 (8)	0.0030 (8)	0.0015 (9)
C1	0.0175 (12)	0.0135 (13)	0.0144 (12)	−0.0004 (10)	0.0053 (10)	0.0009 (10)
C2	0.0181 (12)	0.0145 (14)	0.0201 (14)	0.0003 (10)	0.0053 (10)	0.0030 (11)
C3	0.0185 (12)	0.0136 (14)	0.0215 (14)	−0.0009 (10)	0.0051 (10)	−0.0018 (11)
C4	0.0145 (12)	0.0148 (14)	0.0177 (13)	−0.0020 (10)	0.0041 (10)	−0.0015 (10)
C5	0.0195 (12)	0.0156 (14)	0.0186 (14)	−0.0021 (11)	0.0063 (10)	−0.0052 (11)
C6	0.0188 (13)	0.0201 (15)	0.0132 (12)	−0.0036 (11)	0.0057 (10)	−0.0029 (11)
C7	0.0114 (11)	0.0175 (14)	0.0161 (13)	−0.0023 (10)	0.0037 (10)	0.0012 (10)
C8	0.0179 (12)	0.0231 (16)	0.0124 (12)	−0.0011 (11)	0.0050 (10)	0.0001 (11)
C9	0.0186 (12)	0.0168 (14)	0.0169 (13)	0.0029 (11)	0.0061 (10)	0.0049 (11)
C10	0.0178 (12)	0.0156 (14)	0.0192 (14)	0.0003 (10)	0.0051 (10)	0.0016 (11)
C11	0.0117 (11)	0.0158 (14)	0.0129 (12)	−0.0002 (10)	0.0036 (9)	0.0003 (10)
C12	0.0106 (11)	0.0158 (14)	0.0146 (12)	−0.0014 (10)	0.0029 (9)	−0.0004 (10)
C13	0.0217 (13)	0.0172 (14)	0.0169 (13)	−0.0012 (11)	0.0049 (11)	0.0023 (11)
C14	0.0204 (13)	0.0166 (15)	0.0273 (15)	0.0001 (11)	0.0035 (11)	0.0076 (12)
C15	0.0200 (13)	0.0126 (14)	0.0323 (16)	0.0011 (11)	0.0082 (12)	0.0014 (12)
C16	0.0150 (12)	0.0134 (14)	0.0230 (14)	−0.0016 (10)	0.0055 (10)	−0.0028 (11)
C17	0.0231 (14)	0.0183 (15)	0.0278 (16)	−0.0015 (11)	0.0105 (12)	−0.0087 (12)
C18	0.0211 (13)	0.0265 (17)	0.0200 (14)	−0.0023 (12)	0.0076 (11)	−0.0068 (12)
C19	0.0139 (12)	0.0244 (16)	0.0154 (13)	−0.0031 (11)	0.0070 (10)	−0.0032 (11)
C20	0.0167 (12)	0.0327 (18)	0.0158 (13)	−0.0016 (12)	0.0047 (10)	0.0007 (12)
C21	0.0174 (13)	0.0283 (17)	0.0198 (14)	0.0002 (12)	0.0037 (11)	0.0096 (12)
C22	0.0151 (12)	0.0168 (14)	0.0249 (15)	0.0011 (10)	0.0032 (11)	0.0037 (11)
C23	0.0095 (11)	0.0140 (13)	0.0207 (14)	−0.0018 (10)	0.0050 (10)	0.0001 (11)
C24	0.0126 (11)	0.0153 (14)	0.0185 (13)	−0.0011 (10)	0.0046 (10)	−0.0013 (11)
C25	0.0203 (13)	0.0126 (13)	0.0126 (12)	0.0040 (10)	0.0057 (10)	−0.0022 (10)
C26	0.0320 (15)	0.0161 (15)	0.0144 (13)	0.0025 (12)	0.0074 (11)	−0.0004 (11)
C27	0.0360 (17)	0.0264 (18)	0.0236 (16)	−0.0043 (14)	0.0123 (13)	0.0038 (13)
C28	0.0358 (17)	0.049 (2)	0.0148 (15)	−0.0023 (16)	0.0033 (13)	0.0019 (14)
C29	0.0221 (13)	0.0141 (14)	0.0112 (12)	−0.0001 (10)	0.0064 (10)	−0.0007 (10)
C30	0.0257 (13)	0.0118 (14)	0.0131 (12)	0.0001 (11)	0.0070 (10)	−0.0012 (10)
C31	0.0300 (15)	0.0200 (16)	0.0223 (15)	0.0028 (12)	0.0021 (12)	0.0014 (12)
C32	0.0322 (15)	0.0127 (14)	0.0220 (15)	0.0021 (12)	0.0042 (12)	0.0008 (11)
N5	0.0199 (12)	0.0205 (13)	0.0249 (13)	−0.0021 (10)	0.0027 (10)	0.0013 (10)
O5	0.0235 (10)	0.0409 (14)	0.0254 (12)	−0.0094 (10)	0.0079 (9)	−0.0029 (10)
O6	0.0219 (11)	0.0613 (18)	0.0318 (13)	−0.0054 (11)	0.0121 (10)	−0.0084 (12)
O7	0.0180 (10)	0.0423 (15)	0.0346 (13)	0.0063 (10)	−0.0014 (9)	−0.0056 (11)
N6	0.0287 (13)	0.0129 (12)	0.0192 (12)	0.0035 (10)	0.0059 (10)	0.0011 (10)
O8	0.0217 (10)	0.0276 (12)	0.0204 (10)	−0.0028 (9)	0.0013 (8)	0.0010 (9)
O9	0.0585 (15)	0.0297 (13)	0.0170 (11)	0.0008 (11)	0.0099 (10)	0.0075 (9)
O10	0.0257 (10)	0.0229 (12)	0.0325 (12)	−0.0071 (9)	−0.0003 (9)	0.0062 (9)
O3W	0.0227 (10)	0.0271 (12)	0.0234 (11)	−0.0011 (9)	0.0065 (8)	0.0033 (9)
O4W	0.0298 (11)	0.0450 (15)	0.0206 (11)	−0.0054 (10)	0.0050 (9)	−0.0043 (10)

### Geometric parameters (Å, °)

**Table:**

Cu1—O4	1.9446 (18)	C13—C14	1.396 (4)
Cu1—O1	1.9641 (19)	C13—H13A	0.93
Cu1—N1	2.014 (2)	C14—C15	1.364 (4)
Cu1—N2	2.018 (2)	C14—H14A	0.93
Cu1—O1W	2.1525 (19)	C15—C16	1.412 (4)
Cu2—O2	1.9440 (19)	C15—H15A	0.93
Cu2—O3	1.956 (2)	C16—C24	1.398 (4)
Cu2—N3	2.008 (2)	C16—C17	1.436 (4)
Cu2—N4	2.019 (2)	C17—C18	1.361 (4)
Cu2—O2W	2.1538 (18)	C17—H17A	0.93
O1—C29	1.259 (3)	C18—C19	1.427 (4)
O2—C29	1.266 (3)	C18—H18A	0.93
O3—C25	1.263 (3)	C19—C20	1.404 (4)
O4—C25	1.261 (3)	C19—C23	1.406 (4)
O1W—H1W1	0.85	C20—C21	1.371 (4)
O1W—H2W1	0.85	C20—H20A	0.93
O2W—H1W2	0.85	C21—C22	1.394 (4)
O2W—H2W2	0.85	C21—H21A	0.93
N1—C1	1.337 (3)	C22—H22A	0.93
N1—C12	1.357 (3)	C23—C24	1.424 (4)
N2—C10	1.325 (3)	C25—C26	1.504 (4)
N2—C11	1.364 (3)	C26—C28	1.358 (4)
N3—C13	1.331 (3)	C26—C27	1.459 (4)
N3—C24	1.361 (3)	C27—H27A	0.96
N4—C22	1.334 (3)	C27—H27B	0.96
N4—C23	1.357 (3)	C27—H27C	0.96
C1—C2	1.405 (4)	C28—H28A	0.93
C1—H1A	0.93	C28—H28B	0.93
C2—C3	1.365 (4)	C29—C30	1.495 (4)
C2—H2A	0.93	C30—C32	1.381 (4)
C3—C4	1.414 (4)	C30—C31	1.446 (4)
C3—H3A	0.93	C31—H31A	0.96
C4—C12	1.401 (4)	C31—H31B	0.96
C4—C5	1.431 (4)	C31—H31C	0.96
C5—C6	1.359 (4)	C32—H32A	0.93
C5—H5A	0.93	C32—H32B	0.93
C6—C7	1.434 (4)	N5—O6	1.225 (3)
C6—H6A	0.93	N5—O7	1.245 (3)
C7—C11	1.399 (4)	N5—O5	1.278 (3)
C7—C8	1.410 (4)	N6—O8	1.248 (3)
C8—C9	1.375 (4)	N6—O10	1.249 (3)
C8—H8A	0.93	N6—O9	1.251 (3)
C9—C10	1.402 (4)	O3W—H1W3	0.86
C9—H9A	0.93	O3W—H2W3	0.91
C10—H10A	0.93	O4W—H1W4	0.93
C11—C12	1.436 (4)	O4W—H2W4	0.84
			
O4—Cu1—O1	95.60 (8)	N1—C12—C11	116.4 (2)
O4—Cu1—N1	91.08 (8)	C4—C12—C11	119.7 (2)
O1—Cu1—N1	159.63 (8)	N3—C13—C14	122.1 (3)
O4—Cu1—N2	172.86 (8)	N3—C13—H13A	118.9
O1—Cu1—N2	90.85 (8)	C14—C13—H13A	118.9
N1—Cu1—N2	81.82 (9)	C15—C14—C13	119.9 (3)
O4—Cu1—O1W	90.17 (7)	C15—C14—H14A	120.1
O1—Cu1—O1W	90.99 (8)	C13—C14—H14A	120.1
N1—Cu1—O1W	108.26 (8)	C14—C15—C16	119.6 (3)
N2—Cu1—O1W	92.77 (8)	C14—C15—H15A	120.2
O2—Cu2—O3	94.59 (8)	C16—C15—H15A	120.2
O2—Cu2—N3	174.38 (9)	C24—C16—C15	117.0 (3)
O3—Cu2—N3	90.33 (9)	C24—C16—C17	118.3 (3)
O2—Cu2—N4	92.74 (9)	C15—C16—C17	124.8 (3)
O3—Cu2—N4	159.13 (8)	C18—C17—C16	121.1 (3)
N3—Cu2—N4	81.71 (9)	C18—C17—H17A	119.4
O2—Cu2—O2W	92.09 (8)	C16—C17—H17A	119.4
O3—Cu2—O2W	97.23 (7)	C17—C18—C19	121.2 (3)
N3—Cu2—O2W	89.97 (8)	C17—C18—H18A	119.4
N4—Cu2—O2W	102.00 (8)	C19—C18—H18A	119.4
C29—O1—Cu1	133.72 (18)	C20—C19—C23	116.5 (3)
C29—O2—Cu2	126.17 (18)	C20—C19—C18	125.0 (3)
C25—O3—Cu2	126.70 (17)	C23—C19—C18	118.5 (3)
C25—O4—Cu1	131.56 (18)	C21—C20—C19	120.1 (3)
Cu1—O1W—H1W1	147.0	C21—C20—H20A	120.0
Cu1—O1W—H2W1	98.9	C19—C20—H20A	120.0
H1W1—O1W—H2W1	107.6	C20—C21—C22	119.6 (3)
Cu2—O2W—H1W2	99.0	C20—C21—H21A	120.2
Cu2—O2W—H2W2	99.1	C22—C21—H21A	120.2
H1W2—O2W—H2W2	104.0	N4—C22—C21	122.2 (3)
C1—N1—C12	117.8 (2)	N4—C22—H22A	118.9
C1—N1—Cu1	129.24 (19)	C21—C22—H22A	118.9
C12—N1—Cu1	112.87 (17)	N4—C23—C19	123.4 (2)
C10—N2—C11	118.1 (2)	N4—C23—C24	116.5 (2)
C10—N2—Cu1	129.25 (19)	C19—C23—C24	120.1 (2)
C11—N2—Cu1	112.66 (17)	N3—C24—C16	123.1 (2)
C13—N3—C24	118.3 (2)	N3—C24—C23	116.3 (2)
C13—N3—Cu2	128.63 (19)	C16—C24—C23	120.6 (2)
C24—N3—Cu2	112.89 (17)	O4—C25—O3	125.3 (2)
C22—N4—C23	118.2 (2)	O4—C25—C26	116.8 (2)
C22—N4—Cu2	129.2 (2)	O3—C25—C26	117.8 (2)
C23—N4—Cu2	112.52 (17)	C28—C26—C27	123.4 (3)
N1—C1—C2	122.1 (2)	C28—C26—C25	118.6 (3)
N1—C1—H1A	118.9	C27—C26—C25	117.8 (2)
C2—C1—H1A	118.9	C26—C27—H27A	109.5
C3—C2—C1	119.9 (3)	C26—C27—H27B	109.5
C3—C2—H2A	120.1	H27A—C27—H27B	109.5
C1—C2—H2A	120.1	C26—C27—H27C	109.5
C2—C3—C4	119.6 (3)	H27A—C27—H27C	109.5
C2—C3—H3A	120.2	H27B—C27—H27C	109.5
C4—C3—H3A	120.2	C26—C28—H28A	120.0
C12—C4—C3	116.7 (2)	C26—C28—H28B	120.0
C12—C4—C5	119.0 (2)	H28A—C28—H28B	120.0
C3—C4—C5	124.3 (3)	O1—C29—O2	124.9 (3)
C6—C5—C4	121.2 (3)	O1—C29—C30	117.8 (2)
C6—C5—H5A	119.4	O2—C29—C30	117.3 (2)
C4—C5—H5A	119.4	C32—C30—C31	123.0 (3)
C5—C6—C7	120.8 (3)	C32—C30—C29	118.1 (2)
C5—C6—H6A	119.6	C31—C30—C29	118.8 (2)
C7—C6—H6A	119.6	C30—C31—H31A	109.5
C11—C7—C8	116.9 (2)	C30—C31—H31B	109.5
C11—C7—C6	118.9 (2)	H31A—C31—H31B	109.5
C8—C7—C6	124.2 (2)	C30—C31—H31C	109.5
C9—C8—C7	119.2 (3)	H31A—C31—H31C	109.5
C9—C8—H8A	120.4	H31B—C31—H31C	109.5
C7—C8—H8A	120.4	C30—C32—H32A	120.0
C8—C9—C10	120.0 (3)	C30—C32—H32B	120.0
C8—C9—H9A	120.0	H32A—C32—H32B	120.0
C10—C9—H9A	120.0	O6—N5—O7	122.2 (3)
N2—C10—C9	122.2 (3)	O6—N5—O5	119.1 (2)
N2—C10—H10A	118.9	O7—N5—O5	118.6 (2)
C9—C10—H10A	118.9	O8—N6—O10	119.9 (2)
N2—C11—C7	123.7 (2)	O8—N6—O9	119.9 (2)
N2—C11—C12	116.1 (2)	O10—N6—O9	120.2 (2)
C7—C11—C12	120.2 (2)	H1W3—O3W—H2W3	96.1
N1—C12—C4	123.8 (2)	H1W4—O4W—H2W4	136.4
			
O4—Cu1—O1—C29	−84.4 (2)	C6—C7—C11—C12	2.0 (4)
N1—Cu1—O1—C29	24.2 (4)	C1—N1—C12—C4	1.8 (4)
N2—Cu1—O1—C29	92.6 (2)	Cu1—N1—C12—C4	−175.84 (19)
O1W—Cu1—O1—C29	−174.7 (2)	C1—N1—C12—C11	−178.7 (2)
O3—Cu2—O2—C29	75.1 (2)	Cu1—N1—C12—C11	3.7 (3)
N4—Cu2—O2—C29	−85.4 (2)	C3—C4—C12—N1	0.4 (4)
O2W—Cu2—O2—C29	172.5 (2)	C5—C4—C12—N1	−178.6 (2)
O2—Cu2—O3—C25	−95.0 (2)	C3—C4—C12—C11	−179.2 (2)
N3—Cu2—O3—C25	82.3 (2)	C5—C4—C12—C11	1.9 (4)
N4—Cu2—O3—C25	15.3 (4)	N2—C11—C12—N1	−1.6 (3)
O2W—Cu2—O3—C25	172.3 (2)	C7—C11—C12—N1	177.4 (2)
O1—Cu1—O4—C25	67.2 (2)	N2—C11—C12—C4	178.0 (2)
N1—Cu1—O4—C25	−93.5 (2)	C7—C11—C12—C4	−3.1 (4)
O1W—Cu1—O4—C25	158.2 (2)	C24—N3—C13—C14	−0.2 (4)
O4—Cu1—N1—C1	−1.5 (2)	Cu2—N3—C13—C14	175.11 (19)
O1—Cu1—N1—C1	−110.8 (3)	N3—C13—C14—C15	1.4 (4)
N2—Cu1—N1—C1	179.3 (2)	C13—C14—C15—C16	−0.3 (4)
O1W—Cu1—N1—C1	89.1 (2)	C14—C15—C16—C24	−1.7 (4)
O4—Cu1—N1—C12	175.83 (17)	C14—C15—C16—C17	177.9 (3)
O1—Cu1—N1—C12	66.5 (3)	C24—C16—C17—C18	−2.0 (4)
N2—Cu1—N1—C12	−3.41 (16)	C15—C16—C17—C18	178.5 (3)
O1W—Cu1—N1—C12	−93.64 (17)	C16—C17—C18—C19	−2.0 (4)
O1—Cu1—N2—C10	22.9 (2)	C17—C18—C19—C20	−174.9 (3)
N1—Cu1—N2—C10	−176.2 (2)	C17—C18—C19—C23	3.8 (4)
O1W—Cu1—N2—C10	−68.1 (2)	C23—C19—C20—C21	−0.1 (4)
O1—Cu1—N2—C11	−158.36 (17)	C18—C19—C20—C21	178.5 (3)
N1—Cu1—N2—C11	2.55 (17)	C19—C20—C21—C22	1.4 (4)
O1W—Cu1—N2—C11	110.60 (17)	C23—N4—C22—C21	−1.1 (4)
O3—Cu2—N3—C13	23.8 (2)	Cu2—N4—C22—C21	−179.80 (19)
N4—Cu2—N3—C13	−175.5 (2)	C20—C21—C22—N4	−0.8 (4)
O2W—Cu2—N3—C13	−73.4 (2)	C22—N4—C23—C19	2.5 (4)
O3—Cu2—N3—C24	−160.65 (17)	Cu2—N4—C23—C19	−178.57 (19)
N4—Cu2—N3—C24	−0.01 (17)	C22—N4—C23—C24	−176.5 (2)
O2W—Cu2—N3—C24	102.12 (17)	Cu2—N4—C23—C24	2.4 (3)
O2—Cu2—N4—C22	−3.5 (2)	C20—C19—C23—N4	−1.9 (4)
O3—Cu2—N4—C22	−114.0 (3)	C18—C19—C23—N4	179.4 (2)
N3—Cu2—N4—C22	177.5 (2)	C20—C19—C23—C24	177.1 (2)
O2W—Cu2—N4—C22	89.3 (2)	C18—C19—C23—C24	−1.7 (4)
O2—Cu2—N4—C23	177.76 (17)	C13—N3—C24—C16	−2.0 (4)
O3—Cu2—N4—C23	67.2 (3)	Cu2—N3—C24—C16	−178.02 (19)
N3—Cu2—N4—C23	−1.32 (17)	C13—N3—C24—C23	177.4 (2)
O2W—Cu2—N4—C23	−89.52 (17)	Cu2—N3—C24—C23	1.3 (3)
C12—N1—C1—C2	−2.6 (4)	C15—C16—C24—N3	2.9 (4)
Cu1—N1—C1—C2	174.57 (18)	C17—C16—C24—N3	−176.7 (2)
N1—C1—C2—C3	1.3 (4)	C15—C16—C24—C23	−176.4 (2)
C1—C2—C3—C4	1.0 (4)	C17—C16—C24—C23	4.0 (4)
C2—C3—C4—C12	−1.7 (4)	N4—C23—C24—N3	−2.5 (3)
C2—C3—C4—C5	177.2 (2)	C19—C23—C24—N3	178.4 (2)
C12—C4—C5—C6	0.4 (4)	N4—C23—C24—C16	176.8 (2)
C3—C4—C5—C6	−178.5 (3)	C19—C23—C24—C16	−2.2 (4)
C4—C5—C6—C7	−1.5 (4)	Cu1—O4—C25—O3	−5.5 (4)
C5—C6—C7—C11	0.3 (4)	Cu1—O4—C25—C26	173.17 (18)
C5—C6—C7—C8	−179.6 (2)	Cu2—O3—C25—O4	19.3 (4)
C11—C7—C8—C9	0.4 (4)	Cu2—O3—C25—C26	−159.39 (18)
C6—C7—C8—C9	−179.7 (2)	O4—C25—C26—C28	−173.6 (3)
C7—C8—C9—C10	−0.9 (4)	O3—C25—C26—C28	5.2 (4)
C11—N2—C10—C9	0.8 (4)	O4—C25—C26—C27	2.3 (4)
Cu1—N2—C10—C9	179.52 (18)	O3—C25—C26—C27	−178.9 (3)
C8—C9—C10—N2	0.3 (4)	Cu1—O1—C29—O2	13.8 (4)
C10—N2—C11—C7	−1.4 (4)	Cu1—O1—C29—C30	−164.71 (18)
Cu1—N2—C11—C7	179.72 (19)	Cu2—O2—C29—O1	−4.5 (4)
C10—N2—C11—C12	177.6 (2)	Cu2—O2—C29—C30	174.01 (17)
Cu1—N2—C11—C12	−1.3 (3)	O1—C29—C30—C32	6.6 (4)
C8—C7—C11—N2	0.8 (4)	O2—C29—C30—C32	−172.0 (3)
C6—C7—C11—N2	−179.1 (2)	O1—C29—C30—C31	−175.0 (2)
C8—C7—C11—C12	−178.1 (2)	O2—C29—C30—C31	6.4 (4)

### Hydrogen-bond geometry (Å, °)

**Table:**

*D*—H···*A*	*D*—H	H···*A*	*D*···*A*	*D*—H···*A*
O1W—H1W1···O6^i^	0.85	2.42	3.115 (3)	140
O1W—H1W1···O8^i^	0.85	2.32	2.882 (3)	124
O2W—H1W2···O5^ii^	0.85	2.03	2.761 (3)	144
O3W—H1W3···O5	0.86	1.97	2.811 (3)	163
O4W—H1W4···O10^iii^	0.93	2.02	2.807 (3)	142
O1W—H2W1···O3W^i^	0.85	2.19	2.791 (3)	127
O3W—H2W3···O9^iii^	0.91	2.00	2.862 (3)	157
O4W—H2W4···O7^iii^	0.84	2.29	2.860 (3)	125
C1—H1A···O4	0.93	2.56	3.035 (3)	112
C1—H1A···O10^iv^	0.93	2.53	3.247 (3)	134
C3—H3A···O9^v^	0.93	2.37	3.186 (4)	146
C14—H14A···O4W^vi^	0.93	2.52	3.364 (4)	151
C15—H15A···O2W^ii^	0.93	2.49	3.357 (3)	155
C21—H21A···O3^v^	0.93	2.39	3.318 (4)	179
C28—H28B···O3	0.93	2.42	2.747 (4)	100
C32—H32B···O1	0.93	2.42	2.737 (4)	100
C32—H32B···O8^i^	0.93	2.43	3.345 (3)	168

Symmetry codes: (i) −*x*+1, *y*+1/2, −*z*+1/2; (ii) −*x*, *y*+1/2, −*z*+1/2;  (iii) *x*, −*y*+1/2, *z*+1/2; (iv) *x*, *y*+1, *z*; (v) *x*, −*y*+3/2, *z*+1/2; (vi) *x*, −*y*+3/2, *z*−1/2.
